# Supplementary material for: Seroprevalence of Anti-SARS-CoV-2 Antibodies in Cats during Five Waves of COVID-19 Epidemic in Thailand and Correlation with Human Outbreaks
Source: Animals (Basel). 2024 Feb 29;14(5):761. doi: 10.3390/ani14050761 (PMC10930909; doi:10.3390/ani14050761)
Supplement: Supplementary file 1 [file animals-14-00761-s001.zip › animals-2841085-supplementary.pdf]

Supplementary data

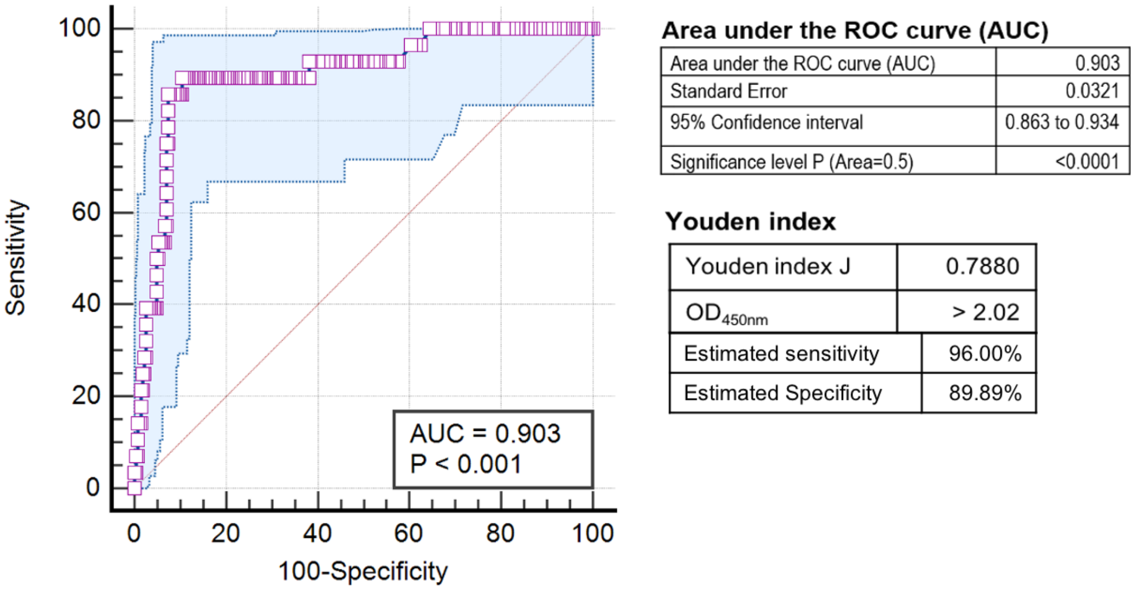

Figure S1 ROC curve and ROC curve analysis with Youden index calculation for determining optimal cutoff value by S1-RBD ELISA test (preliminary population, a part of total population in this study). Samples included in this analysis and calculation was composed of cat sera positive to SARS-CoV-2 (n = 24) and cat sera negative to SARS-CoV-2 (n = 267) confirmed by cPass surrogate viral neutralization.

Table S1. Potentialcross-reactivity of cat sera tested by modified indirect ELISA .

**Cat sera with other diseases**	**S1-RBD ELISA**		**cPass viral neutralization**		**Total N=292**
	**Positive**	**Negative**	**Positive**	**Negative**	
**Healthy cats**	0	20	0	20	20
**Non-infectious diseases**					
Cats in non-outbeak areas*	8	148	0	156	156
Kidney diseases	2	19	0	21	21
Neurological diseases	0	1	0	1	1
Urinary tract diseases	0	13	0	13	13
Respiratory tract diseases	0	4	0	4	4
Heart diseases	0	2	0	2	2
Diseases with unknown causes	0	3	0	3	3
**Infectious diseases**					
Bacterial infection	0	3	0	3	3
Fungal infection	0	3	0	3	3

Parasitic infection	0	1	0	1	1
Bacterial and parasitic infection	0	8	0	8	8
Feline immunodeficiency virus	0	9	0	9	9
Feline leukemia virus (FeLV)	3	16	0	19	19
FIV and FeLV co-infection	0	6	0	6	6
Feline panleukopenia virus (FPV)	1	10	0	11	11
FPV and FeLV co-infection	0	1	0	1	1
Cat flu	0	4	0	4	4
Feline infectious peritonitis (Feline coronavirus)	0	7	0	7	7

*preliminary population, a part of total population in this study
